# IPO5 Mediates EMT and Promotes Esophageal Cancer Development through the RAS-ERK Pathway

**DOI:** 10.1155/2022/6570879

**Published:** 2022-09-09

**Authors:** Meiyu Li, Xiaofei Li, Shujia Chen, Tianai Zhang, Liaoyuan Song, Jiayue Pei, Guoyan Sun, Lianyi Guo

**Affiliations:** The First Affiliated Hospital of Jinzhou Medical University, Jinzhou, China

## Abstract

**Objective:**

In the development of many tumors, IPO5, as a member of the nuclear transporter family, exerts a significant function. Also, IPO5 is used as a therapeutic target for tumors based on some reports. By studying IPO5 expression in esophageal cancer tissues, the mechanism associated with IPO5 improving esophageal cancer development was explored in this study.

**Methods:**

To gain differentially expressed genes, this study utilized mRNA microarray and TCGA database for comprehensive analysis of esophageal cancer tissues and normal esophageal cancer tissues, and then the differentially expressed gene IPO5 was screened by us. To assess esophageal cancer patients' prognosis, this study also applied the Kaplan-Meier analysis, and we also conducted the GSEA enrichment analysis to investigate IPO5-related signaling pathways. This study performed TISIDB and TIMER online analysis tools to study the correlation between IPO5 and immune regulation and infiltration. We took specimens of esophageal cancer from patients and detected the expression of IPO5 in tumor and normal tissues by immunohistochemistry. The IPO5 gene-silenced esophageal cancer cell model was constructed by lentivirus transfection. Through the Transwell invasion assay, CCK-8 assay, and cell scratch assay, this study investigated the effects of IPO5 on cell propagation, invasion, and transfer. What is more, we identified the influences of IPO5 on the cell cycle through flow cytometry and established a subcutaneous tumor-forming model in nude mice. Immunohistochemistry was used to verify the expression of KI-67, and this study detected the modifications of cell pathway-related proteins using Western blot and applied EMT-related proteins to explain the mechanism of esophageal cancer induced by IPO5.

**Results:**

According to database survival analysis, IPO5 high-expression patients had shorter disease-free survival than IPO5 low-expression patients. Compared to normal tissues, the IPO5 expression in cancer tissues was significantly higher in clinical trials (*P* < 0.05). Through TISIDB and TIMER database studies, we found that IPO5 could affect immune regulation, and the age of IPO5 expression grows with the increase of immune infiltration level. The IPO5 expression in esophageal cancer cells was higher than normal, especially in ECA109 and OE33 cells (*P* < 0.01). After knocking out IPO5 gene expression, cell proliferation capacity and invasion capacity were reduced (*P* < 0.05) and decreased (*P* < 0.01) in the IPO5-interfered group rather than the negative control group. The growth cycle of esophageal carcinoma cells was arrested in the G2/M phase after IPO5 gene silencing (*P* < 0.01). Tumor-forming experiments in nude mice confirmed that after IPO5 deletion, the tumor shrank, the expression of KI67 decreased, the downstream protein expression level of the RAS pathway decreased after sh-IPO5 interference (*P* < 0.01), and the level of EMT marker delined (*P* < 0.05).

**Conclusion:**

In esophageal cancer, IPO5 is highly expressed and correlates with survival rate. Esophageal cancer cell growth and migration were significantly affected by the inhibition of IPO5 in vitro and in vivo. IPO5 mediates EMT using the RAS-ERK signaling pathway activation and promotes esophageal cancer cell development in vivo and in vitro.

## 1. Introduction

Presently, esophageal cancer (EC) is the sixth dominant inducement of death resulting from cancer [1] and is the 8th most common cancer in the world [2]. One of the countries with a high incidence of esophageal cancer includes China [2]. EC patients have no symptoms in the early stage, but when a series of symptoms such as swallowing difficulty and wasting appear, they have quietly entered the middle and late stage. In China, patients' 5-year survival rate in the middle and advanced stages is only 15%-25% [3, 4]. Therefore, to reduce the incidence of EC and the main cause of death in China, going deeper into the biological mechanism of the early occurrence and rapid progression of esophageal cancer, studying more excellent gene-targeted treatment, and available, and effective methods will exert a more profound and great influence.

One of the most fundamental pathways for cell metabolism and survival is nuclear plasma transport. Its transport dysfunction leads to many diseases including cancer [5]. Nuclear transporters (karyopherins) are key regulatory molecules of nuclear plasma transport, including input protein and output protein5. The protein constructed by the IPO5 gene comes from the import protein *β* family, which is a type of nuclear transporter and is located in the region of chromosome 13q32, which is involved in the process of transporter protein [6]. Current studies on IPO5 are limited and are mainly focused on the following fields: Wnt pathway activity is involved in the development of various benign and malignant cancers, IPO5 interacts with IQGAP1, a regulatory factor of *β* -catenin, and loss of IPO5 reduces the expression of Wnt target genes during early embryogenesis [7]; human herpes virus 8 (HHV-8) can bind to IPO5mRNA, and the mechanism may be to induce the production of miRNA, leading to the pathogenesis of Kaposi's sarcoma [8]. IPO5 mediates RASAl2 nuclear transport to enhance the propagation and shift [9]. There are currently two reported examples of preliminary studies on molecular inhibitors of nuclear import proteins. Intraperitoneal administration of INI-43 significantly inhibited the growth of subcutaneous xenograft esophageal and cervical neoplasms by targeting Kpn*β*1 [10]. SINE of CRM1/XPO1, a target of chronic lymphocytic leukemia, could effectively covalently bind cysteine residues in XPO1 cargo binding tank to treat the disease and is being tested in clinical trials targeting a wide range of malignancies [11]. However, the study and treatment of IPO5 in malignant tumors are still few.

The RAS-ERK pathway exerts a significant function when adjusting pivotal cellular activities, covering proliferation, survival, differentiation, and motility, and is a cascade that translates the extracellular environment into intracellular signaling, encompassing a range of biochemical processes [12, 13]. It has been suggested that the biological effects of GTPase activator protein 1 (IQGAP1) on tumor cells can be induced by regulating the response of the RAS signaling pathway [14] and binding to actin is contained in diverse processes like cell binding, propagation, circulate, and transfer [15–20]. Therefore, it is inferred that, through adjusting the RAS-ERK signaling pathway, IPO5 may promote esophageal cancer development.

We screened the gene database in the early stage of the experiment, for differentially expressed genes in esophageal cancer. Meanwhile, immunohistochemistry confirmed that in esophageal carcinoma tissues, IPO5 was expressed highly. The results showed that the changes in the esophageal cancer cell cycle could be significantly affected by the low expression of IPO5, and the growth of esophageal cancer was inhibited, possibly through the activation of the RAS-ERK pathway, which leads to epithelial-mesenchymal transformation and thus causes the occurrence of esophageal cancer. We may discover a new biomarker of esophageal cancer, providing a new molecular mechanism for further related development.

## 2. Materials and Methods

### 2.1. Main Materials

The First Affiliated Hospital of Jinzhou Medical University provided cancerous and normal tissues for six patients with esophageal cancer. All patients did not have preoperative radiation therapy or chemotherapy, and paraffin embedding was performed after the cancer tissue was removed. The ethics committee of the affiliated hospital approved all clinical specimen collection, and the patients themselves gave their consent. Esophageal cancer cells ECA109 were purchased from the Chinese Typical Culture Treasure Center. We purchased 8 BALB/C nude mice from Weitonglihua, Beijing. They were 4 weeks old and cultivated in the SPF Animal Experimental Center of Jinzhou Medical University. From Shanghai Enzyme Institute, this study purchased OE33, OE19, TE-1, and normal esophageal epithelial cells HEEC purchased from Mingzhou Biology, Ningbo. Transfection control sequence (sh-NC) and IPO5-interfering lentivirus sequence (sh-IPO5) were purchased from Shanghai Jima Biological Company. The Takara reverse transcription kit was purchased from Japan; ABI Real-time Fluorescence quantitative kit was purchased from the USA. The qRT-PCR primer sequences of IPO5 and GAPDH were obtained from the NCBI database, and the primers were synthesized by the Invitrogen Company. RPMI1640 medium and the freezer-storage solution were purchased from the Wako Company. This study bought fetal bovine serum and 0.25% trypsin-EDTA solution from the Sigma Company. And BD matrix glue and Transwell chamber were purchased from Corning, USA; the CCK-8 kit and BCA kit were purchased from Biyuntian; the cell cycle kit was purchased from Fuyuan Biology; efficient RIPA lysate was purchased from Beijing Solebao; 5 × loading buffer, GAPDH antibody, and CDK6 antibody were purchased from Beijing Bairuji; IPO5 antibodies were purchased from Santa Company; CKD4, CyclinD1, MEK, P-MEK, ERK, P-ERK, and anti-rabbit, and anti-rat antibodies were purchased from Boolsen in Beijing; AKT, P-AKT, N-Cad, Slug, and Vimentin were purchased from Wanlei Biological. The immunohistochemistry kit is available from ZSGB-BIO.

### 2.2. Methods

#### 2.2.1. Data Collection

From the National Center for Biotechnology Information comprehensive gene expression database (GEO) (https://www.ncbi.cn), we obtained the expression profiles of GSE104958 and GSE131027. The raw data of both groups were preprocessed using the multiarray averaging and SVA packages to eliminate batch effects. SVA package preprocessing and multiarray averaging were utilized to eliminate batch influences and combine both sets of unprocessed data. To compare esophageal cancer tissue samples with normal esophageal cancer tissue samples, we applied TCGA database R programming language limma package, with *P* < 0.05 and |logFC|1 or 1 as the threshold values and with the intersection of genetic variants, and finally identified 15 DCGs (differentially expressed genes).

#### 2.2.2. R2 Database

R2 (https://hgserver1.amc.nl/cgi-bin/r2/main.cgi), which is a gene function prediction database, discusses the expression differences in these two esophageal cancer tissues.

#### 2.2.3. GEPIA Database

Gene Expression Profile Interaction Analysis covers 33 cancers (GEPIA Analysis) (http://gepia.cancer-pku.cn/index.html). It is a network-based tool used to deanalyze RNA sequencing expression data with 8,587 normal samples and 9,736 tumor samples from TCGA and GTEx covered. This database was used to explore the expression differences between esophageal carcinoma and normal tissues.

#### 2.2.4. Gene Set Enrichment Analysis (GSEA) Molecular Characterization Database

Available genes were collected from (http://software.broadinstitute.org/gsea/msigdb). GSEA was used to deexamine the correlation between signaling pathways and IPO5 expression.

#### 2.2.5. The TIMER Database

Web server updates, analysis, and visualization of tumor immunity to its association with other tumor's molecular and clinical traits were used as TIMER 2.0 (http://timer.cistrome.org/). TIMER can well evaluate the level of immune aggression in TCGA cohort and find correlations between immune aggression, gene expression, mutations, and survival traits. In other words, extensive analysis and tumor-infiltrating immune cells visualization are empowered by the TIMER 2.0 web server.

#### 2.2.6. Cell Culture

We placed HEEC, ECA109, TE-1, OE33, and OE19 in an RPMI1640 medium containing 10% fetal bovine serum and incubated the cells in a cell culture incubator at 37°C with 5% CO2.

#### 2.2.7. Cell Transfection

We placed ECA109 and OE33 cells in 24-well plates and shifted them to the infected venom after 1 day. Cells were cultured for 2 days after transfection according to the transfection instructions and observed. When the cells grew well and the number of plate layers reached 90% or more, the cells were collected for subsequent experiments.

#### 2.2.8. The Expression of IPO5 RNA in the Collected Samples Was Detected by QRT-PCR

Total RNA was extracted with TRIzol lysis reagent, and we synthesized cDNA with a cDNA reverse transcription kit. We assayed QRT-PCR through the ABI real-time fluorescence quantification kit. We used the 2^-*ΔΔ*Ct^ method to calculate GAPDH as an internal reference for mRNA. The upstream primer of IPO5 is 5′-GAGAAGCCTTTCCAGGACCC-3′, and the downstream primer sequence is 5′-AGTGTTGTTGCTGGTTCCCA-3′. The upstream primer sequence of GAPDH is 5′-AATGGGCAGCCGTTAGGAAA-3′, and the downstream primer sequence is 5′-GCGCCCAATACGACCAAATC-3′.

#### 2.2.9. Cell Scratch Experiment

The back of the petri dish was evenly marked with a marker pen. After the cells were covered with the bottom of the 6-well plate, three vertical lines were evenly marked with the head of a gun. After gently rinsing with PBS, RPMI1640 was added for culture and incubated in a cell incubator. The ImageJ software was used for analysis.

#### 2.2.10. Transwell Invasion Experiment

We cultured the cells for 48 h after digestion and then diluted them to 5 × 10^4^ cells/ml and set them aside. A 24-well plate was taken to wrap the cells with matrix glue and place them in the incubator for 1 hour. After glue fixation, 200 *μ*l of diluted cell suspension was added. We added 600 *μ*l of 20% PBS medium to each lower chamber. We gently swabbed the upper luminal cells with a cotton swab after 32 h of incubation. We counted the number of cells invading and crossing the lumen after fixation staining under the microscope.

#### 2.2.11. CCK-8 Cell Proliferation

First, ECA109 cells and OE33 cells were inoculated into 96-well plates at an inoculation density of 3000 cells/well. The transfection was detected at 1, 2, 3, 4, and 5 days after transfection. Add CCK-8 reagent to each well, and incubate it in a cell culture box for 1 hour. Keep it out of the light. Then the absorbance at 450 nm was measured by a microplate reader, and the cell growth curve was drawn.

#### 2.2.12. Flow Cytometry Detection Cell Cycle

We added 70% precooled ethanol after cell digestion, PBS washing, and rehanging and placed it overnight in a 4°C refrigerator. Ethanol attached to the cell surface was washed with PBS, and PI solution containing RNA enzyme was used to stain for half an hour (incubators at 37°C were kept away from light). Flow cytometry was used for detection.

#### 2.2.13. Tumorigenesis Experiment in Nude Mice

We split the nude rats randomly into 2 groups of 4 rats each. ECA109 cell control group and positive group with good growth after treatment were selected to make single-cell suspension and counted. RPMI1640 and matrix glue were mixed in a ratio of 1 : 2, and cells were diluted and inoculated in the right axilla of nude mice, with the inoculation amount of about 5 × 10^6^ cells. After the subcutaneous nodules were observed to be visible to the naked eyes, we estimated the tumor size weekly over approximately 41 days. We exfoliated subcutaneous tumors and photographed them after the death of nude mice. We performed Western blot to measure the EMT-related protein expression.

#### 2.2.14. The Expression of KI67 and IPO5 Was Detected by Immunohistochemistry

According to the steps of dewaxing, rehydration, antigen repair, sealing, incubation of primary antibody, incubation of secondary antibody, DAB color, nuclear redyeing, dehydration, sealing, and finally observation and statistics under the microscope, the nuclei of KI67 were brown granules. Four fields were randomly selected under the microscope, and the positive rate of each field = number of positive cells/total cells × 100%. The statistical index of IPO5 was that the proportion of positive staining tissues was <10%, 10%-30%, 30%-50%, and >50%, and then 0-3 points were counted successively. According to the intensity of staining, the tissues were divided into no staining, light yellow, dark yellow, and dark brown, and 0-3 points were counted successively. The two scores were multiplied to obtain the total score.

#### 2.2.15. Western Blot Test

With the total protein concentration, the protein test cell loading buffer diluted the protein. After electrophoresis, transferring, and closing operations, the primary antibody was manually diluted according to the primary antibody at 4 degrees in the freezer overnight. PBS was repeatedly added and shaken on a shaker for 10 minutes and washed three times. Secondary antibody was added, incubated in a shaker for 1 hour according to the instructions, and enhanced with three more washes of PBS at room temperature.

### 2.3. Statistical Analysis

We analyzed the statistics using the R (V.3.5.1) and SPSS software. We drew pictures going with GraphPad Prism 7. Through an independent *t*-test, we compared continuous data between the two groups. *P* < 0.05 was used as the difference was statistically significant. One-way ANOVA was applied to compare data between multiple groups. Chi-square was applied to test for declassified information.

## 3. Results

### 3.1. Screening of Differential Genes (DEGs)

**Figure 1:**
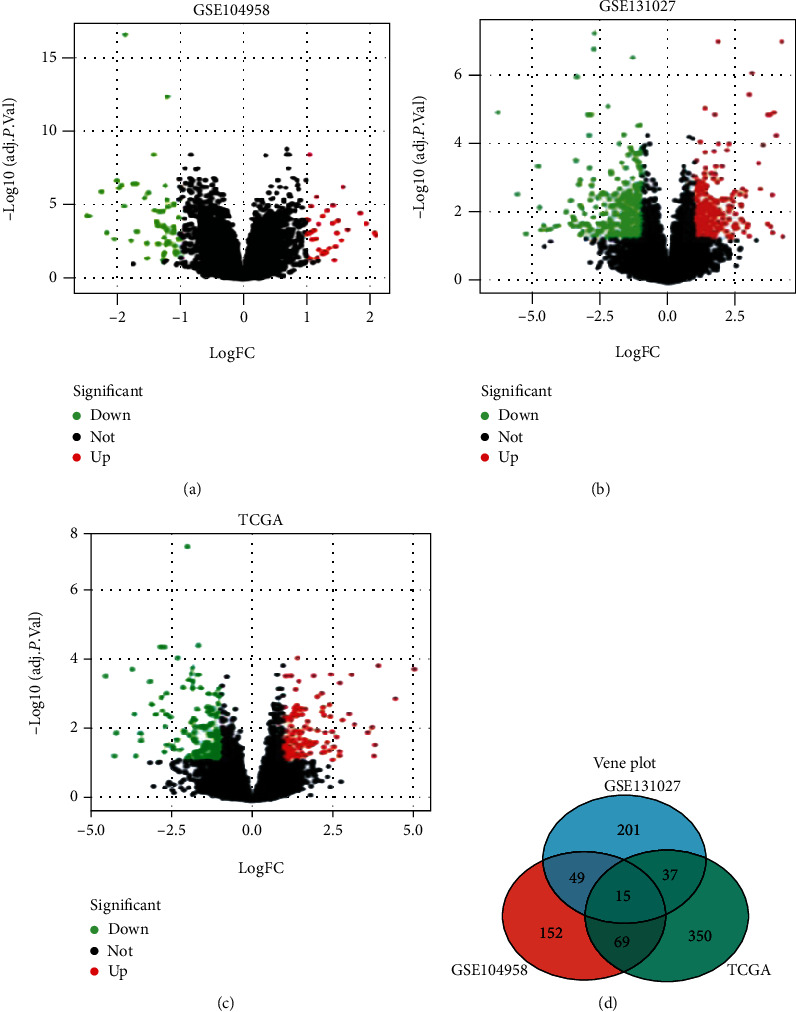
Differentially expressed genes between esophageal cancer and normal tissues. (a–c) Volcanic map of differentially expressed genes between esophageal cancer tissues and normal esophageal tissues. IPO5 is highly expressed in esophageal cancer tissues. (d) Venn diagram distribution of differentially expressed genes from GSE104958 and GSE131027 arrays and TCGA database. As shown in the figure, a total of 15 genes showed specific differential expression in three datasets.

DEG (i.e., GSE104958 and GSE131027) was analyzed using the limma software package after pretreatment and elimination of batch impact. Fifteen DEGs were obtained with *P* < 0.05 and |logFC| 1 or 1 as the threshold and intersected with TCGA database DCG (Figure 1(d)). According to |logFC|, the values of the volcano show a rise in red marker genes and green marker genes (Figures 1(a)–1(c)). Ultimately, the IPO5 gene was screened and found to be differentially expressed, in esophageal cancer tissue versus normal tissue.

### 3.2. IPO5 Is a Prognostic Predictor of Esophageal Cancer

**Figure 2:**
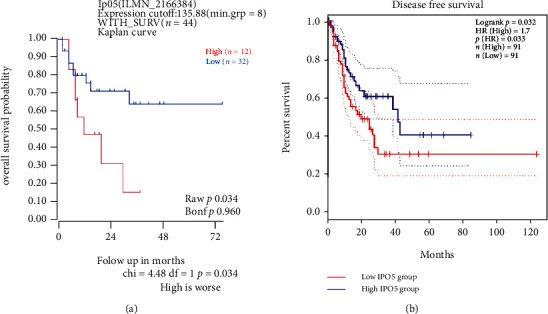
The prognostic value of IPO5 in esophageal cancer. (a) There was a great difference in survival between the two groups with high and low IPO5 expression (*P* = 0.034). (b) The high IPO5 expression group had worse disease-free survival than the low IPO5 expression group (*P* = 0.032).

In the prognosis prediction of esophageal cancer, R2 and GEPIA databases were used to go for survival analysis for determining the functions of IPO5 expression. Compared to patients with low IPO5 expression, this result showed that disease-free survival in high IPO5 patients was shorter with a statistically significant difference (Figure 2). It is indicated that IPO5 may be related to a bad prognosis.

**Figure 3:**
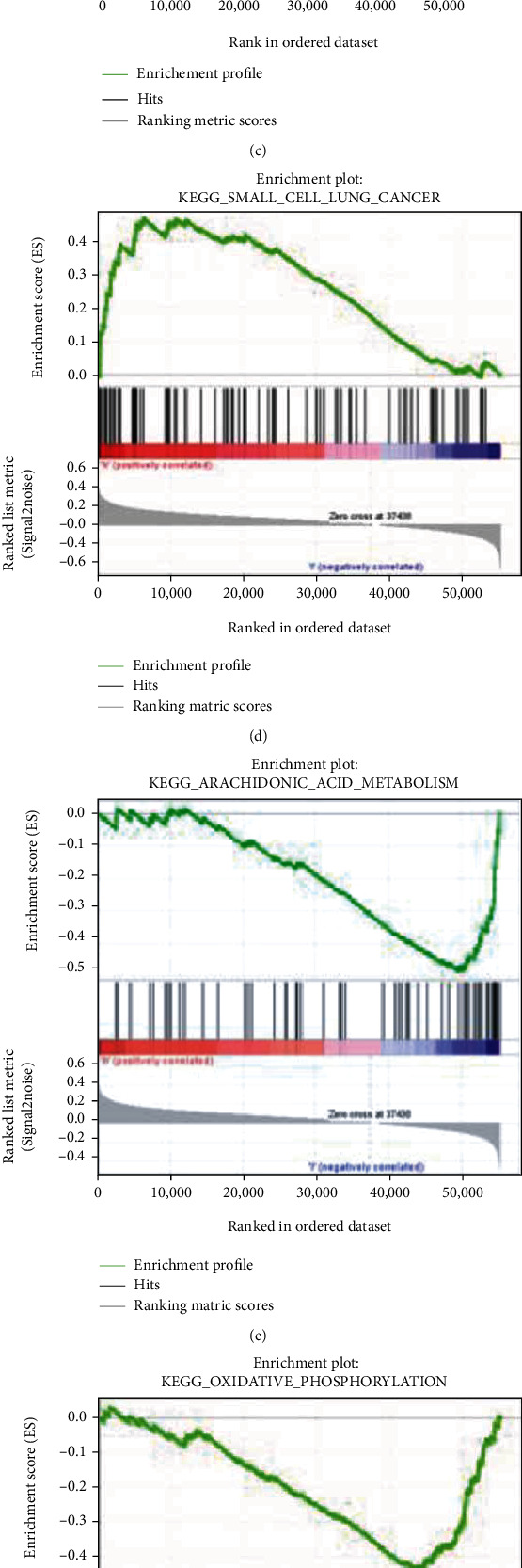
IPO5 enrichment analysis diagram based on GSEA. GSEA presented both group's expression was mainly enriched in (a) basic transcription factors, (b) cell cycle, (c) RNA degradation, (d) small cell lung cancer, (e) oxidative phosphorylation, (f) Parkinson's disease, (g) phenylalanine metabolism, and (h) arachidonic acid metabolism, in 8 pathways. NES: normalized enrichment score; ES: concentration score; FDR: error detection rate.

Based on the analysis of enrichment of MSigDB (c2. Cp. Kegg. V6.2. Symbols. GMT), GSEA results show the distinction between the two groups was statistically significant. The 4 most significantly enriched signaling pathways are in the IPO5 high group. By contrast, in the low-expression group of IPO5, the four most significantly enriched signaling pathways were oxidative phosphorylation, Parkinson's disease, phenylalanine metabolism, and arachidonic acid metabolism (Figure 3).

### 3.3. IPO5 Expression Is Related to the Immune System

Based on earlier research, there is a great correlation between the tumorigenesis and immune system.

**Figure 4:**
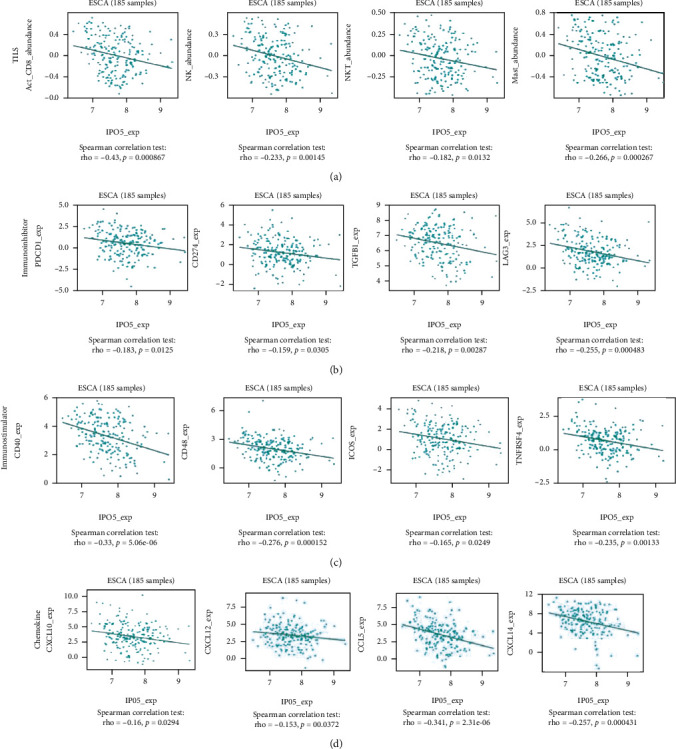
The expression of IPO5 is related to the immune system. IPO5 was significantly correlated with tumor immune lymphocytes, immunosuppressants, immune activators, and chemokines (*P* < 0.001).

As a result, the PO5 effect on immune factors needs to be further investigated. It was discovered a great negative relationship between IPO5 and tumor immune lymphocytes, immunosuppressants, immune activators, and chemokines (*P* < 0.001) (Figure 4).

### 3.4. IPO5 Is Upregulated in Esophageal Carcinoma Cells

**Figure 5:**
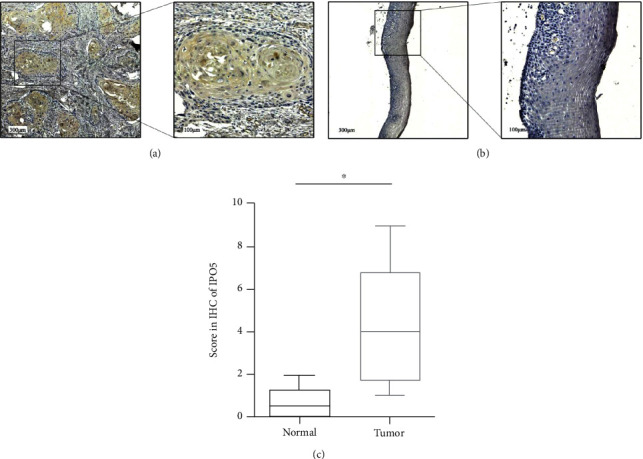
IPO5 expression in 6 patients with esophageal cancer was higher in cancer tissues than in adjacent tissues. (a) Esophageal cancer tissues showed high IPO5 expression. (b) Low expression of IPO5 in normal tissues of esophageal cancer. (c) Increased expression of IPO5 in cancer tissues was calculated by the immunohistochemical score in 6 patients with esophageal cancer (note: ^∗^*P* < 0.05).

In our previous experiment, through applying the database, IPO5 was analyzed as the differentially expressed gene between these two esophageal tissues. Subsequently, we collected postoperative specimens of clinical esophageal cancer patients for immunohistochemical experiments to further verify the above conclusions. Compared with normal esophageal normal tissues next to cancer, based on IPO5 expression in 6 pairs of esophageal cancer tissues and normal tissues, there was a significant upregulation of the expression level of IPO5 in tumor tissues, and cytoplasmic staining was primarily used to detect IPO5 (*P* < 0.05) (Figure 5).

### 3.5. Expression of IPO5 in Various Esophageal Cancer Cells

**Figure 6:**
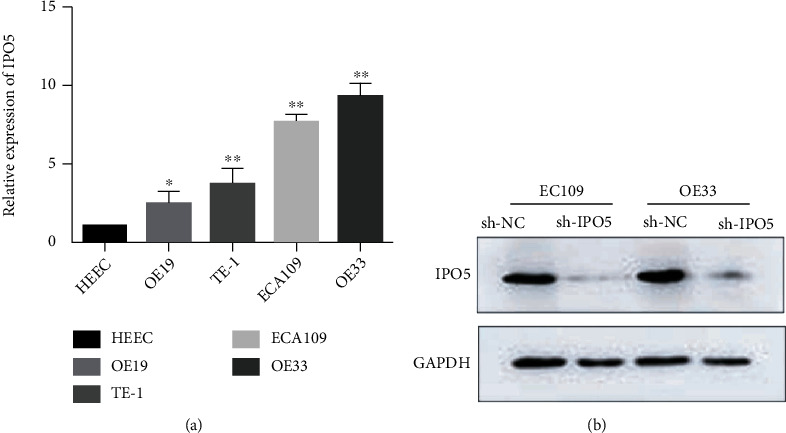
(a) QRT-PCR analysis presented that IPO5 expression in esophageal cancer cell lines was higher than that in normal HEEC esophageal cells, and the highest expression was found in OE33 and ECA109 cells. (b) Western blot assay showed that the expression of IPO5 was significantly downregulated after interference with IPO5 lentivirus by esophageal carcinoma cells (note: ^∗^*P* < 0.05; ^∗∗^*P* < 0.01).

IPO5 expression was measured by QRT-PCR. In esophageal cancer cells, the IPO5 expression was greatly higher than in normal human esophageal epithelial cells (HEEC), especially in ECA-109 and OE33 cells (*P* < 0.01) (Figure 6(a)). Subsequently, Western blot was applied to ECA109 and OE33 cells to verify the transfection effect of IPO5, and it was found that the expression of IPO5 decreased after interference. Therefore, ECA109 and OE33 cell lines were used for the following experiments. This study split cancer cells into sh-IPO5 and sh-NC groups based on whether they were transfected with IPO5 lentivirus or not (Figure 6(b)).

### 3.6. Interference with IPO5 Inhibits the Invasion, Migration, and Proliferation of Esophageal Cancer Cells

**Figure 7:**
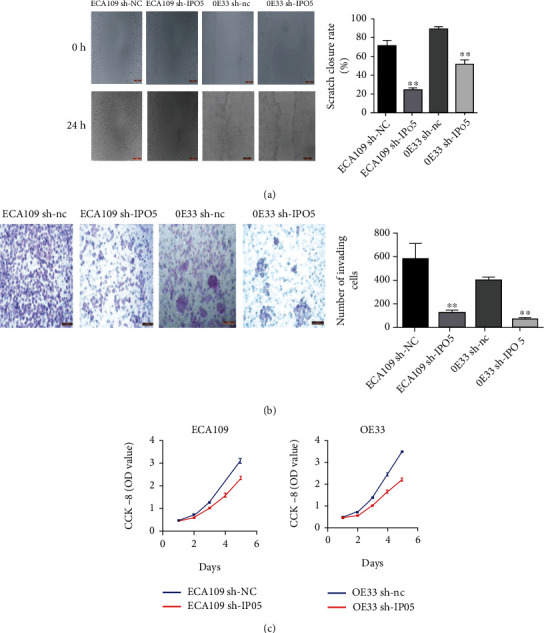
(a) Cell scratch test presented that the closure rate of the sh-IPO5 group was lower than that of the sh-NC group at 24 h. (b) Transwell invasion test presented the number of cells crossing the chamber was increased in the sh-NC group compared with the sh-IPO5 group. (c) CCK-8 analysis of sh-IPO5 reduced the viability of ECA109 and OE33 cells (note: ^∗^*P* < 0.05; ^∗∗^*P* < 0.01).

Figure 7(a) presents that in the scratch test of ECA109 cells and OE33 cells, we found that the ability of the sh-IPO5-positive group to transfer had a declined ground migration capacity (*P* < 0.01) than the control group. As shown in Figure 7(b), it was found that the positive group in the Transwell invasion test has a weaker invasive capacity (*P* < 0.01).  Figure 7(c) presents the CCK-8 experiment, compared with the control group, and we found that with the increase of days, the positive group had a weaker cell proliferation ability (*P* < 0.05). In summary, esophageal cancer cells' reduced migration, invasion, and proliferation can be affected by reduced IPO5 gene expression.

### 3.7. Cell Growth Cycle Was Inhibited after IPO5 Interference

We utilized flow cytometry to measure changes in the cell cycle before and after IPO5 gene silencing. It was discovered that compared with the control group, ECA109 and OE33 cell proportion in the G2/M stage was greatly aggrandized in the sh-IPO5 group.

**Figure 8:**
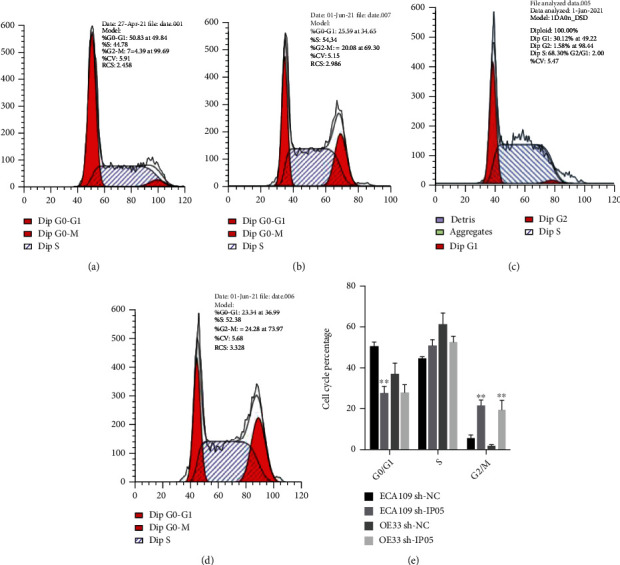
Cell cycle changes before and after IPO5 gene silencing detected by flow cytometry. (a, b) The proportion of G2/M phase increased before and after transfection of ECA109 cells. (c, d) The proportion of G2/M phase in OE33 cells increased before and after transfection. (e) The proportion of ECA109 and OE33 cells in the sh-IPO5 group was significantly higher than that in the sh-NC group in the G2/M phase (note: ^∗∗^*P* < 0.01).

The changes in the cell cycle before and after IPO5 gene silencing were detected by flow cytometry. It was found the ratio of ECA109 and OE33 cells in the G2/M phase was greatly aggrandized in the sh-IPO5 group (*P* < 0.01), indicating interference with IPO5 can promote G2/M phase arrest of esophageal cancer cells (Figure 8).

### 3.8. Tumorigenesis Experiment in Nude Mice

**Figure 9:**
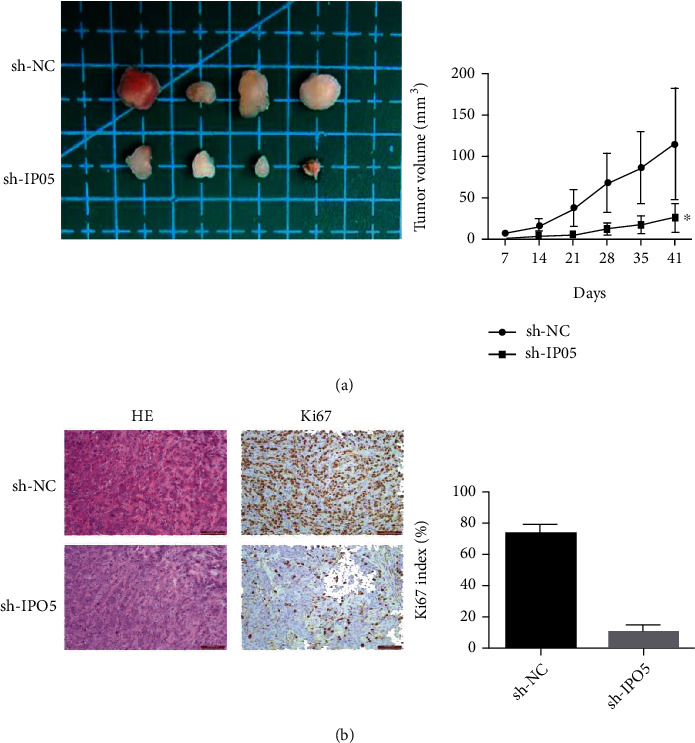
(a) Tumor formation experiment in nude mice presented that the growth rate of nude mice in the positive group was significantly lower than that in the negative control group. (b) Immunohistochemical test presented that KI67 expression in the positive group was significantly lower than that in the negative control group (note: ^∗∗^*P* < 0.01).

The nude mouse model was established and tumor sizes were documented weekly to deeply validate the IPO5 gene role in esophageal cancer. It was observed that tumor growth was greatly slower in the IPO5 knockout group compared to the negative control group (*P* < 0.01) (Figure 9(a)). Subsequently, the KI67expression was verified by immunohistochemistry, and the KI67expression level was found to be significantly reduced in the IPO5 gene silencing group (*P* < 0.01) (Figure 9(b)), suggesting IPO5 had an accelerating effect on esophageal cancer development.

### 3.9. Interference with IPO5 Inhibits Ras Signaling Pathway

**Figure 10:**
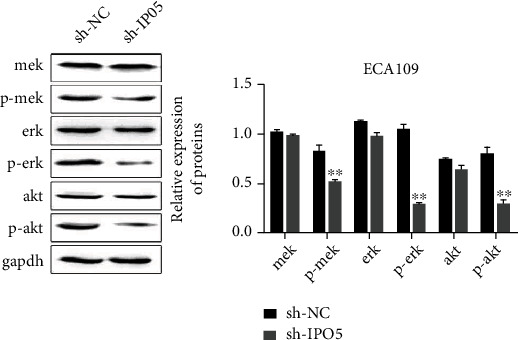
Western blot analysis showed that knockdown of IPO5 gene significantly reduced the expressions of P-MEK, P-ERK, and P-AKT but had no significant effect on the expressions of MEK, ERK, and AKT (note: ^∗∗^*P* < 0.01).

In the sh-NC and sh-IPO5 groups of nude mouse tumors, we examined the expression of RAS pathway-related proteins MEK, P-MEK, ERK, P-ERK, AKT, and P-AKT by Western blot. The findings revealed there was a big downregulation of P-MEK, P-ERK, and P-AKT expressions after interference by IPO5. There were no obvious modifications in total protein expression (*P* < 0.01) (Figure 10), indicating that progression into esophageal cancer can be promoted by IPO5 through the RAS signaling pathway.

### 3.10. Interference with IPO5 Inhibits Epithelial-Mesenchymal Transformation

**Figure 11:**
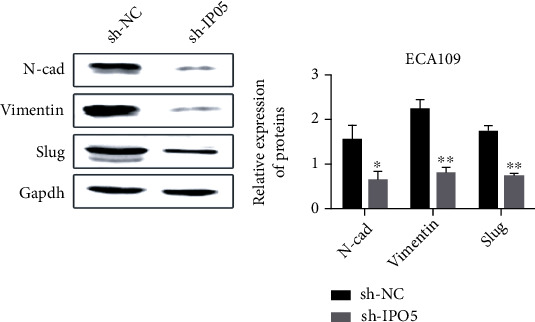
Western blot analysis showed that knockdown of IPO5 gene significantly reduced the expression of N-cad, Vimentin, and Slug (note: ^∗^*P* < 0.05; ^∗∗^*P* < 0.01).

The IPO5 impact on epithelial transformation-related protein expression was identified by Western blot. Compared with the control group, the outcomes revealed that N-cad, Vimentin, and Slug protein expression levels were remarkably reduced in the IPO5 gene silencing group (*P* < 0.05) (Figure 11), suggesting that IPO5 promotes the occurrence of EMT-induced esophageal cancer.

## 4. Discussion

Nowadays, one of the world's deadliest tumors includes malignant tumors of the esophagus. Statistics show that Asia accounts for 80% of the total global incidence of disease about 21 incidence of esophageal cancer, and the symptoms are hardly found at the early stage [21]. Moreover, the advanced cancer patient's quality of life plummeted, and the survival rate is extremely low. However, people now more and more realize the importance of screening in ordinary times, and early diagnosis of esophageal cancer detection index is imminent.

In the process of nuclear plasma transport, karyopherins play an essential role as transport officers, including nuclear input proteins and export proteins [22]. Currently, nuclear transporters have been implicated in cancer progression. XPO-t overexpression is associated with poor outcomes in breast, ovarian, and mesothelioma [23, 24]. According to previous research, there was a relationship between cancer staging and prognosis CAS and overexpression in cancer cells [25]. IPO5 overexpression may occur in bladder cancer, thyroid cancer, breast cancer, malignant melanoma, colorectal cancer, etc. [26, 27]. It has been reported that DDX56 can inhibit the introduction of IRF3 nuclei by destroying the assembly of IRF3-IPO5, thereby inhibiting type I interferon, and thereby regulating antiviral innate immunity [28]. Relevant studies have shown that the expression level of IPO5 is significantly correlated with tumor immune lymphocytes, immunosuppressants, immune activators, and chemokines, which indicates that IPO5 can participate in tumor immune microenvironment and mediate tumor immune response [29, 28]. The reduction of Wnt target genes expression involved in the development of various benign and malignant tumors can be achieved by the deletion of IPO5. Human herpetic virus 8 (HHV-8) binds to IPO5mRNA, leading to Kaposi's sarcoma [8]; IPO5 mediates RASAl2 nuclear transport to accelerate the colorectal cancer cells proliferation and migration [9]. Cell scratching, invasion and proliferation experiments, flow cytometry, and tumorigenesis experiments in nude mice in this study were used to fully demonstrate that the IPO5 gene can improve esophageal cancer proliferation, differentiation, and occurrence. Through a certain pathway, we speculated that IPO5 may accelerate cancer cell propagation.

Activation of the Wnt/*β*-catenin pathway has been considered related to EMT. The classical Wnt pathway inhibits GSK-3*β*-mediated *β*-catenin and plays a role in phosphorylation and degradation. Ning et al. proposed that the Wnt/GSK-3*β*/*β*-catenin pathway explored the change process of S100A4-induced mesenchymal transformation of nasal mucosal epithelial cells from molecular mechanism to cell morphology, which laid a foundation for the systematic analysis of the mechanism of nasal mucosal tissue remodeling and the search for new targets for treatment [30]. The activated AS-ERK signaling pathway is present in many cancers. RAS protein acts as a molecular switch. In the inactive state, and the conversion of GTP to GDP results in the activation of GTPase in RAS genes [31]. Activated GTP-binding RAS further transmits the signal to the downstream RAF-MEK-ERK signal cascade. In turn, various transcription factors in the nucleus are activated, thus affecting cell proliferation and differentiation, promoting cancer invasion and metastasis, etc. [32]. The RAS-ERK pathway is involved in many cancer developments. Research on the RAS-ERK signaling pathway has been increasing in recent years. For example, tumorigenic Sergi-mediated transcription in prostate cells could be adjusted by RAS-ERK and PI3K-AKT signaling pathways differentially [33]. Vemurafenib significantly reduced the proliferation of melanocyte nevus by targeting the RAS-ERK pathway [34]. Modulators of the RAS-ERK pathway can be used as therapeutic targets for thyroid cancer [35]. Inhibition of the RAS-ERK pathway provides a novel therapeutic strategy for gemcitabine-resistant pancreatic cancer [36]. Related literature has shown that GTPase activator protein 1 (IQGAP1) can induce tumor biology based on the regulation of the RAS pathway and participates in diverse cell processes involving cell transfer, propagation, and cycle after binding with actin. At the same time, IPO5 can regulate the transcription of IQGAP1 protein, so we speculated that IPO5 can promote the progression of esophageal cancer through the RAS pathway. To verify this hypothesis, we measured low IPO5 expression influence on RAS pathway-related proteins through Western blot, and it was found that when IPO5 was knocked down, the expression of phosphorylated proteins P-ERK, P-MEK, and P-AKT was significantly reduced, while the total expression of ERK, MEK, and AKT was not significantly changed, hence the above corollary.

Epithelial-mesenchymal transition (EMT) is a process in which epithelial cells acquire mesenchymal characteristics. In cancer, EMT is related to tumor occurrence, invasion, metastasis, and drug resistance. Recently, it has been proved that EMT is not a binary process but occurs through different cell states, and different EMT states have different regulatory mechanisms for cancer. Proliferation, plasticity, invasion, and metastasis are related to different EMT states. We summarized the role of transcriptional and epigenetic landscapes, gene regulatory networks, and their surrounding niches in controlling the transition through different EMT states [37, 38]. A growing body of literature highlights the link between cancer-related EMT, which stimulates cancer cells to produce proinflammatory factors, and chronic inflammation. In contrast, the getting of EMT-like features in cancer cells can be facilitated by inflammatory mediators such as oxidative stress, soluble factors, or hypoxia. Therefore, the two phenomena may reinforce each other, making the body more prone to the formation of metastatic lesions [39]. In our experiment, we found that IPO5 was significantly negatively correlated with tumor immune lymphocytes, immunosuppressants, immune activators, and chemokines. Western blot also confirmed that IPO5 could promote the epithelial-mesenchymal transformation of esophageal cancer. It can be concluded from the above discussion that IPO5 can be used as a new biological marker to give a novel perspective for future cancer diagnosis and therapy. However, as a kind of nuclear transporter, IPO5 can cooperate with related proteins to enter the nucleus to regulate cancer-related signaling pathways, and our research mechanism in this aspect is limited. As a result of the small sample size collected clinically, some errors might exist, which we will continue to improve in future experiments.

## 5. Conclusion

In conclusion, the gene that triggers esophageal cancer is IPO5, and EMT mediated through the RAS pathway accelerated the progression of esophageal cancer and may be a promising targeted therapy to eradicate early-stage esophageal cancer.

## Data Availability

The datasets used and/or analyzed during the current study are available from the corresponding authors on reasonable request.
